# Supplementary material for: Predicting visual function by interpreting a neuronal wiring diagram
Source: Nature. 2024 Oct 2;634(8032):113–23. doi: 10.1038/s41586-024-07953-5 (PMC11446822; doi:10.1038/s41586-024-07953-5)
Supplement: Supplementary file 6 — CRF and ERF predictions for individual TmY4 and TmY9 cells. Analogous to Supplementary Data 3, but for TmY target types. Shown are the top four monosynaptic pathways, the strongest pathway passing through each of the top ten intermediary types (ranking from Extended Data Fig. 7), and the trisynaptic pathway Tm1–TmY–Dm3–TmY (see the section entitled Prediction of spatial normalization). [file 41586_2024_7953_MOESM6_ESM.zip › DataS4/TmY9q/720575940623886921.pdf]

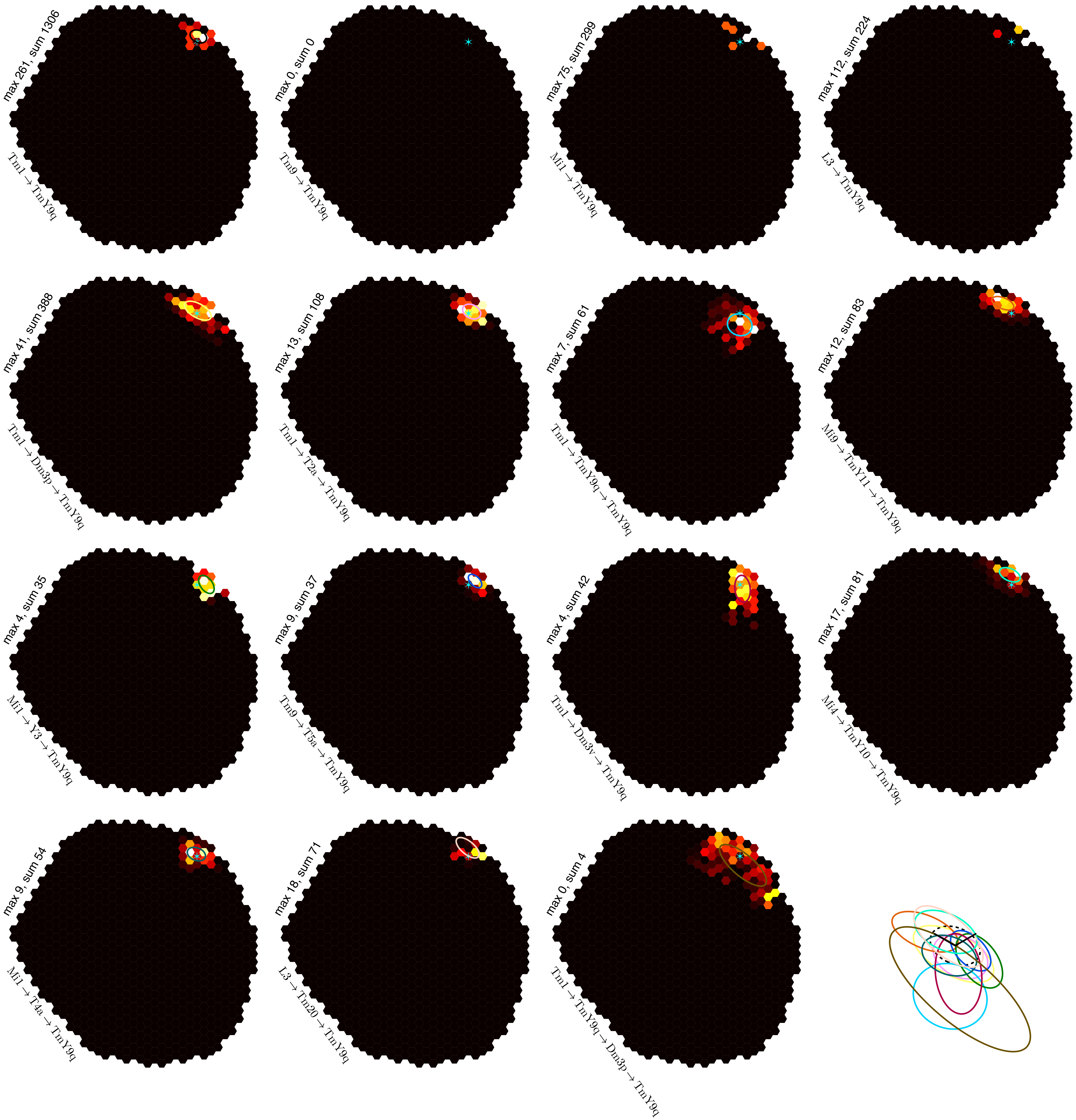

max 261, sum 1306
Tm1 → TmY9q
max 0, sum 0
Tm9 → TmY9q
max 75, sum 299
Mi1 → TmY9q
max 112, sum 224
L3 → TmY9q
max 41, sum 388
Tm1 → Dm3p → TmY9q
max 13, sum 108
Tm1 → T2a → TmY9q
max 7, sum 61
Tm1 → TmY9q → TmY9q
max 12, sum 83
Mi9 → TmY11 → TmY9q
max 4, sum 35
Mi1 → Y3 → TmY9q
max 9, sum 37
Tm9 → T5a → TmY9q
max 4, sum 42
Tm1 → Dm3v → TmY9q
max 17, sum 81
Mi4 → TmY10 → TmY9q
max 9, sum 54
Mi1 → T4a → TmY9q
max 18, sum 71
L3 → Tm20 → TmY9q
max 0, sum 4
Tm1 → TmY9q → Dm3p → TmY9q
